# Long-Term *In Vitro* Passaging Had a Negligible Effect on Extracellular Vesicles Released by *Leishmania amazonensis* and Induced Protective Immune Response in BALB/c Mice

**DOI:** 10.1155/2021/7809637

**Published:** 2021-12-24

**Authors:** Talita Vieira Dupin, Natasha Ferraz de Campos Reis, Elizabeth Cristina Perez, Rodrigo Pedro Soares, Ana Claudia Torrecilhas, Patricia Xander

**Affiliations:** ^1^Laboratório de Imunologia Celular e Bioquímica de Fungos e Protozoários, Departamento de Ciências Farmacêuticas, Universidade Federal de São Paulo, Campus Diadema, Diadema, Brazil; ^2^Pós Graduação em Patologia Ambiental e Experimental, Universidade Paulista, São Paulo, Brazil; ^3^Instituto René Rachou/FIOCRUZ, Belo Horizonte, Brazil

## Abstract

Depending on *Leishmania* species and the presence/absence of virulence factors, *Leishmania* extracellular vesicles (EVs) can differently stimulate host immune cells. This work is aimed at characterizing and evaluating the protective role of EVs released by *Leishmania amazonensis* promastigotes under different maintenance conditions. Initially, using a control strain, we standardized 26°C as the best release temperature to obtain EVs with a potential protective role in the experimental leishmaniasis model. Then, long-term (LT-P) promastigotes of *L. amazonensis* were obtained after long-term *in vitro* culture (100 *in vitro* passages). *In vivo*-derived (IVD-P) promastigotes of *L. amazonensis* were selected after 3 consecutive experimental infections in BALB/c mice. Those strains developed similar lesion sizes except for IVD-P at 8 weeks post infection. No differences in EV production were detected in both strains. However, the presence of LPG between LT-P and IVD-P EVs was different. Groups of mice immunized with EVs emulsified in the adjuvant and challenged with IVD-P parasites showed decreased lesion size and parasitic load compared with the nonimmunized groups. The immunization regimen with two doses showed high IFN-*γ* and IgG2a titers in challenged mice with either IVD-P or LT-P EVs. IL-4 and IL-10 were detected in immunized mice, suggesting a mixed Th1/Th2 profile. EVs released by either IVD-P or LT-P induced a partial protective effect in an immunization model. Thus, our results uncover a potential protective role of EVs from *L. amazonensis* for cutaneous leishmaniasis. Moreover, long-term maintenance under *in vitro* conditions did not seem to affect EV release and their immunization properties in mice.

## 1. Introduction

Leishmaniasis is a neglected disease distributed in tropical and subtropical regions, especially in developing countries [1]. It is estimated that 700,000 to 1 million new cases and 26,000 to 65,000 deaths annually are due to infection by the parasite [1]. The cutaneous form is the most common among leishmaniasis, with most cases reported in the Americas, Mediterranean region, Middle East, and Central Asia [1, 2]. Currently, leishmaniasis has no efficacious vaccine for humans, there are few therapeutic options for treatment, and the control of vector and reservoir hosts are not practical or eco-friendly [3].

Macrophages are important cells in response to *Leishmania* infection [4]. After phagocytosis, the formation and maturation of phagolysosome-containing parasites are crucial for parasite killing [5]. Classically activated macrophages (M1) are able to destroy *Leishmania* parasites since they exhibit high microbicidal capacity with the production of high levels of reactive oxygen species (ROS) and nitric oxide (NO) [6, 7]. On the other hand, macrophages can provide an environment for *Leishmania* replication. Alternatively, activated macrophages (M2) show an anti-inflammatory profile contributing to tissue regeneration and wound healing [6]. The role of distinct macrophage populations in the control of *Leishmania* infection still needs to be better clarified.

The control of the parasite by the mammalian host is related to developing an IFN-*γ*-producing CD4^+^ Th1 profile [4], whereas susceptibility is associated to a Th2 response [4]. IFN-*γ* cytokine has an important effect on macrophages since it activates these cells to produce high amounts of microbicidal molecules (such as NO), contributing to parasite elimination [4]. Thus, approaches targeting an effective immune response are promising strategies for treatment/prevention of leishmaniasis.

Different methodologies have been assessed for developing an effective human vaccine for leishmaniasis [8]. Potential candidate vaccines have explored the use of dead parasites [9], genetically modified parasites [10, 11], or molecular systems, such as viruses expressing *Leishmania* genes [12, 13], recombinant proteins [14], and plasmid DNA-based vaccines [15]. Despite efforts, there is still no approved vaccine for the prevention of human leishmaniasis.

Extracellular vesicles (EVs) are an innovative route for delivering antigenic material, providing a promising alternative for vaccine development [16]. They can deliver proteins, lipids, nucleic acids, DNA, and RNA from one cell to another [17, 18]. They may modulate immune responses, facilitating infection, among other functions [19]. EVs released by promastigote forms of *L. amazonensis* activated macrophages via TLR2/4 and by NF-*κ*B translocation, and this effect was higher than those elicited by *Leishmania infantum* and *Leishmania braziliensis* EVs [20–22]. *In vitro*, EVs from *L. amazonensis* promastigotes induced IL-6 and IL-10 by murine bone marrow-derived macrophages (BMDM) [20]. Similar effects were observed in THP-1 macrophage cell human lineages that increased the production of NO, TNF-*α*, IL-6, and IL-10 after treatment with EVs released by *L. amazonensis* promastigotes [21]. On the other hand, EVs of *L. amazonensis* amastigotes released in the parasitophorous vacuole of macrophages led to an inhibition of NO production by infected macrophages [22]. Thus, further studies are needed to better understand the role of these EVs on macrophage activation.

Currently, it is still unknown if long-term *in vitro* culture will affect EVs released by *L. amazonensis* and their immunization properties, especially during vaccination protocols. As a part of a wider study on *L. amazonensis*, EVs from parasites bearing different maintenance conditions were evaluated and characterized. BALB/c mice were immunized with these EVs and their protective effects were evaluated. This work brings new possibilities for using parasite EVs to modulate the immune system, enabling the development of interesting alternatives for treating and preventing cutaneous leishmaniasis.

## 2. Materials and Methods

### 2.1. Animals

Female BALB/c mice (6 to 8 weeks old) were purchased from the Center for the Development of Experimental Models for Medicine and Biology (CEDEME) (Universidade Federal de São Paulo (UNIFESP), São Paulo, SP, Brazil). Animals were maintained under specific pathogen-free conditions recommended by the National Council for Control Animal Experimentation (CONCEA) of Brazil. During the experimental period, mice were fed with a sterilized commercial rodent diet and filtered water *ad libitum*. Animals were housed in microisolator cages in a room with a maintained 12 h light/dark cycle. The parameter temperature, humidity, and air quality were monitored and controlled. Animal procedures were approved by the Committee on Ethics of Animal Experiments (CEUA) of UNIFESP under protocol number 2068080319. All efforts were made to minimize the animals' suffering.

### 2.2. Parasites

The *L. amazonensis* reference strain (MHOM/BR/1973/M2269) was kindly provided by Dra. Clara Lucia Barbieri (UNIFESP). Promastigotes were cultured in M199 medium (Gibco, Life Technologies Brand, Grand Island, NY, USA) supplemented with 4.2 mM sodium bicarbonate, 4.2 mM HEPES, 1 mM adenine, 5 mg/mL hemin (bovine type I) (Sigma, St. Louis, MO, USA), and 10% inactivated fetal bovine serum (FBS) (Gibco). Parasites were cultured at 26°C until they reach the stationary growth phase and used in experimental infection of mice or to obtain EVs.

### 2.3. Experimental Infection of BALB/c Mice with *L. amazonensis* Promastigotes

Parasites were cultured as promastigotes until the stationary phase. After washing with sterile PBS, parasites were resuspended at a concentration of 1 × 10^6^ parasites/20 *μ*L and then subcutaneously inoculated at the right hind footpad in BALB/c mice. The diameter of foot lesions was evaluated weekly by monitoring the induration diameter with a digital caliper. After 6–8 weeks of infection, the entire paws were aseptically removed from euthanatized mice and individually homogenized in M199 medium. The parasite burden was evaluated by the limiting dilution method [23].

### 2.4. Long-Term (LT-P) and *In Vivo*-Derived (IVD-P) Promastigotes

**Figure 1:**
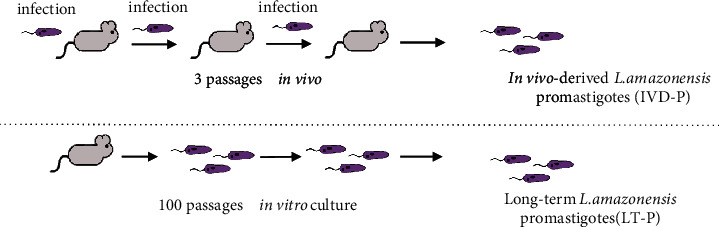
Scheme for obtaining *L. amazonensis* parasites with different virulence profiles (IVD-P and LT-P).

Long-term (LT-P) promastigotes of *L. amazonensis* were obtained after long-term *in vitro* culture with M199 medium plus 10% FBS. *In vitro* passages were performed every five days until completing 100 *in vitro* passages (Figure 1). *In vivo*-derived (IVD-P) promastigotes of *L. amazonensis* were selected after subsequent parasite recovery from 3 consecutive experimental infections in BALB/c mice. Parasites were recovered from lesions in the footpad (Figure 1). Aliquots containing the same passage in culture were frozen for conducting the proposed experiments. For freezing, 2 × 10^7^/mL promastigotes were added in a solution with fresh medium with 5% (*v*/*v*) dimethyl sulfoxide (DMSO). Stocks of 1 mL were maintained in liquid nitrogen.

### 2.5. Isolation and Characterization of EVs Released by *L. amazonensis* Promastigotes

A total of 1 × 10^8^*L. amazonensis* (control strain with intermediate virulence profile) [20, 24] promastigotes from stationary cultures were incubated with 1 mL of RPMI 1640 medium plus 2% D-dextrose for 4 h [20, 21]. Parasites were incubated at 26 or 37°C (26°C corresponds to the vector temperature; 37°C corresponds to the host vertebrate temperature) for 4 h [20]. Afterwards, the supernatants were subjected to serial centrifugation, as follows: 500 × g for 10 min at 4°C, 1,500 × g for 10 min at 4°C, 10,000 × g for 10 min at 4°C, and 2 ultracentrifugations at 100,000 × g for 1 h at 4°C. Then, the pellets were diluted in sterile PBS [20]. EVs from LT-P or IVD-P were obtained from 1 × 10^8^ promastigotes of LT-P or IVD-P from stationary cultures incubated in 1 mL of RPMI 1640 medium plus 2% D-dextrose for 4 h at 26°C. After supernatant collection, EVs were obtained by serial centrifugations and ultracentrifugations, as described above.

Size and concentrations of EVs were analyzed by nanoparticle tracking analysis (NTA) in a NanoSight Equipment NS300 Instrument (Malvern, Instruments Ltd., Malvern, United Kingdom). The apparatus was equipped with a CCD camera and a 405 nm laser. Each sample was diluted 10- to 100-fold in filtered PBS and captured in triplicate for 1 min (20 frames per second) at 20°C. The camera level was set to 14, and the threshold was always the same. The results were analyzed in the NTA software (version 2.3 build 0017) [20]. Protein concentration was determined by using a Micro BCA Protein Assay Kit (Thermo Scientific, Waltham, MA, United States), according to the manufacturer's procedures.

Enzyme-linked immunosorbent assay (ELISA) was performed with EVs to evaluate the presence of gp63 or LPG. ELISA was performed in 96-well plates sensitized overnight at 4°C with 4 *µ*g/mL of each EVs (corresponding to 5.4 × 10^6^ particles of LT-P and 3.2 × 10^6^ particles of IVD-P) released from LT-P or IVD-P. Blocking was performed with 5% nonfat dry milk in PBS for 1 h at 37°C. Monoclonal antibody CA7AE (1: 500) [25] anti-LPG or monoclonal antibody anti-gp63 (1 : 500) (mouse mAb #235) were added, and the plates were incubated for 1 h at 37°C (both mAbs kindly provided by Rodrigo P. Soares, Instituto René Rachou/FioCruz, MG, Brazil). Plates were washed with 0.05% PBS-Tween 20 and incubated with anti-IgG conjugated to peroxidase (SeraCare, KPL, Milford, MA, United States) (1 : 10,000). The reaction was revealed with TMB substrate solution (Pierce Biotechnology, Thermo Fisher, Rockford, IL, United States) and stopped with 2 N H_2_SO_4_. Absorbance was analyzed at 450 nm in an ELISA reader (BioTek, Winooski, VT, United States).

### 2.6. BALB/c Mice Immunization with EVs

First, animals were intraperitoneally immunized with 4 *µ*g of EVs (corresponding to 5.4 × 10^6^ of particles) obtained from *L. amazonensis* promastigote reference strain incubated at 26 or 37°C without adjuvant. Control groups were immunized with PBS.

To evaluate the protective potential of EVs from LT-P and IVD-P, BALB/c mice were immunized intraperitoneally with 4 *µ*g of EVs (corresponding to 5.4 × 10^6^ particles of LT-P and 3.2 × 10^6^ particles of IVD-P). The Alum adjuvant (Adj) (Thermo) was added in the immunization protocols with EVs from LT-P and IVD-P to improve the immune response and to induce the antibody production. Control groups were immunized with alum adjuvant or PBS. Each group was composed of 5–8 animals. Immunizations were carried out at 15-day intervals.

**Figure 2:**
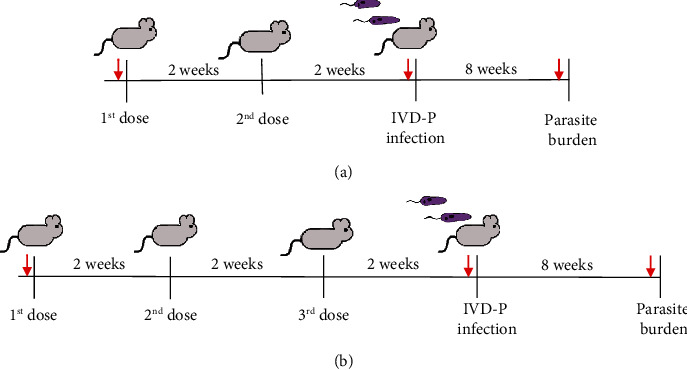
Immunization protocols of BALB/c mice with EVs released by LT-P or IVD-P *L. amazonensis* promastigotes. After 2 weeks of the last immunization, BALB/c mice were infected in the hind footpad with 1 × 10^6^ IVD-P. (a) Protocol with 2 doses and (b) protocol with 3 doses. Red arrows represent blood collection (before the first immunization, before the challenge with the parasites, and at the euthanasia).

After 2 weeks of the last immunization, mice were infected in the footpad with 1 × 10^6^*L. amazonensis* stationary promastigotes of IVD-P. Infection was monitored for 6–8 weeks by measuring the edema with a caliper. Parasite burden was determined by the limiting dilution method. Animals were bled before immunizations, before parasite challenge and during euthanasia.  Figure 2 shows the workflow performed for immunizations and blood sample collection.  Figure 2(a) shows the immunization protocol for 2 doses, and Figure 2(b) represents the design for 3 doses.

### 2.7. Evaluation of Antibody Production

Sera of all mice were collected before the first immunization, before the challenge with the parasites, and at the time of euthanasia. After immunization protocol, the anti-EV antibody production was evaluated by ELISA. Therefore, ninety-six well plates (Costar, Corning Incorporated, NY, United States) were sensitized with 50 *μ*L/well of *L. amazonensis* EVs with a final concentration of 6 *μ*g/mL (corresponding to 8.1 × 10^6^ particles/mL of LT-P and 4.8 × 10^6^ particles of IVD-P). After 6–8 weeks of challenge with the parasites, sera were tested by ELISA to evaluate the presence of anti-*Leishmania* antibodies. Thus, 50 *μ*L of *L. amazonensis* promastigotes' total extract was added in 96-well plates at a 10 *μ*g/mL concentration. Total extract was obtained with parasites submitted to 10 freeze-thaw cycles. Plates sensitized with EVs or total extract were incubated for 1 h at 37°C. Then, the remaining sites were blocked with 1% PBS-BSA solution for 1 h at 37° C. Wells were washed with 0.1% PBS-Tween 20. The serum samples were diluted in PBS-BSA 1% at 1 : 50 dilution, and subsequently, serial dilution was performed. The plates were incubated for 1 h at 37°C, and then, the anti-IgG1 or anti-IgG2a (Thermo Fisher) conjugated to peroxidase diluted 1 : 10,000 (KPL) were added. The plates were incubated for 1 h at 37°C and revealed with TMB. The reaction was stopped after the appearance of color with 100 *μ*L/well of 4 NH_2_SO_4_. The results were evaluated by spectrophotometric reading in an ELISA reader (BioTek) at 450 nm.

### 2.8. Cytokine Production

Spleen cells derived from animals immunized with EVs from LT-P or IVD-P were aseptically removed and used to evaluate cytokine production. Cells from mice immunized with adjuvant were used as negative control. Splenocytes were added in 96-well culture plates with a concentration of 1 × 10^6^ cells/well. Cells were incubated for 5 days with medium alone, EVs (25 *µ*g/mL), or concanavalin-A (positive control). Supernatants were collected, stored at −80°C, and used to evaluate cytokine production.

Th1/Th2/Th17 BD cytometric bead array (CBA) (BD Biosciences) was used to evaluate cytokines in the supernatants of spleen cells from immunized animals. All procedures were performed according to the manufacturer. BD FACS Accuri C6 flow cytometer (BD Biosciences) was used for the acquisitions, and a total of 2,400 events were acquired for each preparation. Data were analyzed by FCAP ArrayTM software (BD Bioscience). Cytokine concentrations were determined based on the calibration curves constructed with known cytokine standards.

### 2.9. Statistical Analysis

All statistical analyzes were performed using the GraphPad Prism 8.0 software (GraphPad Software, La Jolla, CA, United States). The results are shown as the mean ± standard deviation (SD). Data that assumed a Gaussian distribution were analyzed with parametric tests. Therefore, comparison of multiple groups was carried out with the analysis of variance (ANOVA) followed by the Tukey post test. Two-way ANOVA followed by the Sidak post test was used to analyze a bunch of independent comparisons. Student's *t*-tests were used to evaluate the comparison between two groups. Nonparametric data were analyzed with Mann-Whitney for comparing two groups, and Kruskal-Wallis followed by the Dunn test was used to compare three or more groups. ^∗^*P* values < 0.05 were considered significant.

## 3. Results

### 3.1. EVs Obtained from *L. amazonensis* Promastigotes Incubated at Different Temperatures Showed Partial Protection in Immunized BALB/c Mice

**Figure 3:**
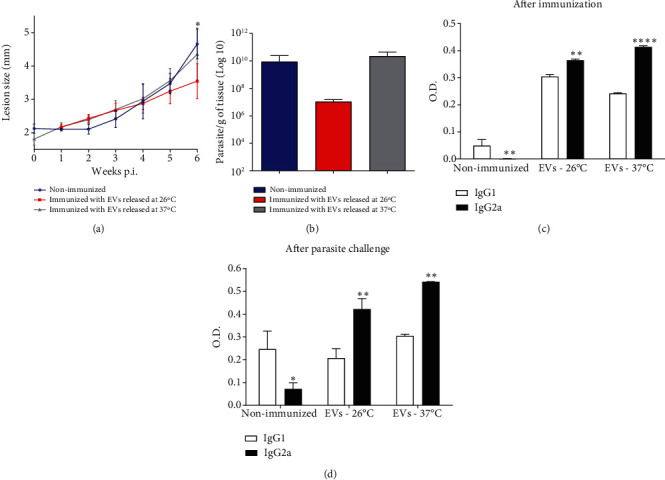
Immunization with EVs released by promastigotes of the reference strain of *L. amazonensis* incubated at 26 or 37°C. BALB/c mice were handled with the 2 doses protocol of EVs from *L. amazonensis* reference strain. (a) The lesion size (millimeter (mm)) measurements. Each point represents the average of the individual measurement (*n* = 5). ANOVA followed by a post hoc Tukey's test was performed ^∗^*P* < 0.05. Nonimmunized mice compared with mice immunized with EVs obtained at 26°C. (b) Parasite load (*n* = 5). Bars denote the average of 5 measurements, and error bars denote the SD. ANOVA followed by a post hoc Tukey's test. (c) Production of IgG1 and IgG2a isotype antibody anti-EVs after two doses of EV immunizations. (d) Measurement of the IgG1 and IgG2a isotype antitotal extract of *L. amazonensis* in mice immunized and infected with the parasite. Bars represent the average of 5 measurements, and error bars show the SD. Two-way ANOVA followed by a post hoc Sidak test (^∗^*P* < 0.05,  ^∗∗^*P* < 0.01, and^∗∗∗∗^*P* < 0.001 IgG1 versus IgG2a in each group). EVs −26°C—mice immunized twice with EVs obtained from *L. amazonensis* promastigotes incubated for 4 h at 26°C; EVs −37°C—mice immunized with 2 doses of EVs obtained from *L. amazonensis* promastigotes incubated for 4 h at 37°C. Data are representative of 3 independent experiments.

BALB/c mice were immunized with 2 doses of EVs from *L. amazonensis*. EVs were obtained from parasites incubated at 26 or 37°C for 4 h [20]. After 2 weeks of the last immunization, mice were subcutaneously challenged in the footpad with 1 × 10^6^*L. amazonensis* promastigotes. The lesion size increased in all challenged groups, but the group of mice immunized with EVs obtained at 26°C showed a significant smaller lesion in comparison to the nonimmunized group (*P* < 0.05, Figure 3(a)). After 6 weeks, the mice were euthanized, and the infected hind footpads were removed. The parasite load showed a decrease (statistically nonsignificant) in the group immunized with EVs obtained at 26°C compared with the other groups (Figure 3(b)).

The analysis of IgG1 and IgG2a antibody subtypes contributes to assessing Th1/Th2 response profiles since IgG1 titers are related to the Th2 profile while IgG2a titers are associated with Th1. Thus, the sera collected after the second immunization with EVs were used to detect IgG1 or IgG2a isotype antibody anti-EVs. After the second immunization with EVs, a significant increase in the levels of IgG2a in comparison with the levels of IgG1 was detected (^∗^*P* < 0.05;  ^∗∗^*P* < 0.01;  ^∗∗∗∗^*P* < 0.001) (Figure 3(c)). After 6 weeks of infection, the levels of IgG1 and IgG2a antitotal extract of *L. amazonensis* promastigotes were evaluated. After 6 weeks of challenge with the parasites, animals showed higher levels of IgG2a compared with IgG1 (^∗^*P* < 0.05; ^∗∗^*P* < 0.01) (Figure 3(d)), except the nonimmunized infected group that showed an increase in IgG1 levels (Figure 3(d)). These results suggest that immunized animals showed a higher induction of Th1 response, compared with the nonimmunized group.

Altogether, these data demonstrated that EVs released by promastigotes of *L. amazonensis* can induce a Th1 response. In addition, the immunization with EVs from parasites incubated at 26°C led to a significant reduction in the lesion size (^∗^*P* < 0.05) and a decrease (though not significant) in the parasite load. Thus, we established this temperature (26°C) as ideal to obtain EVs to use in the subsequent immunization protocols.

### 3.2. Obtaining *L. amazonensis* Parasites with Different Virulence Profiles

**Figure 4:**
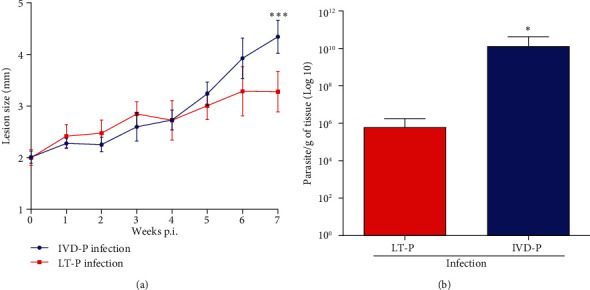
Evaluation of BALB/c mice infection with IVD-P or LT-P. (a) Lesion size (millimeter (mm)) measurements (*n* = 5). Each point represents the average of the measurements (*n* = 5). Student's *t*-test ^∗∗∗^*P* < 0.001. (b) Parasite load in the hind footpad (*n* = 5). Bars denote the average of 5 measurements, and error bars denote the SD. Mann-Whitney test (^∗^*P* < 0.05). Data are representative of 2 independent experiments.

EVs can carry distinct molecules and virulence factors that can differently stimulate the immune response. Thus, we generated *L. amazonensis* with different virulence profiles (LT-P and IVD-P) to evaluate their EVs in immunization protocols. First, we confirmed the differences in the infection profile of LT-P and IVD-P by experimental cutaneous leishmaniasis. After 7 weeks of infection, the lesion size of mice infected with the LT-P was statistically smaller than that of the group infected with the IVD-P on the 7^th^ week (^∗∗∗^*P* < 0.001) (Figure 4(a)). The parasitic load was significantly lower in the group infected with LT-P *L. amazonensis* compared with the group infected with the virulent parasite (^∗^*P* < 0.05) (Figure 4(b)).

### 3.3. Characterization of EVs from LT-P and IVD-P

**Figure 5:**
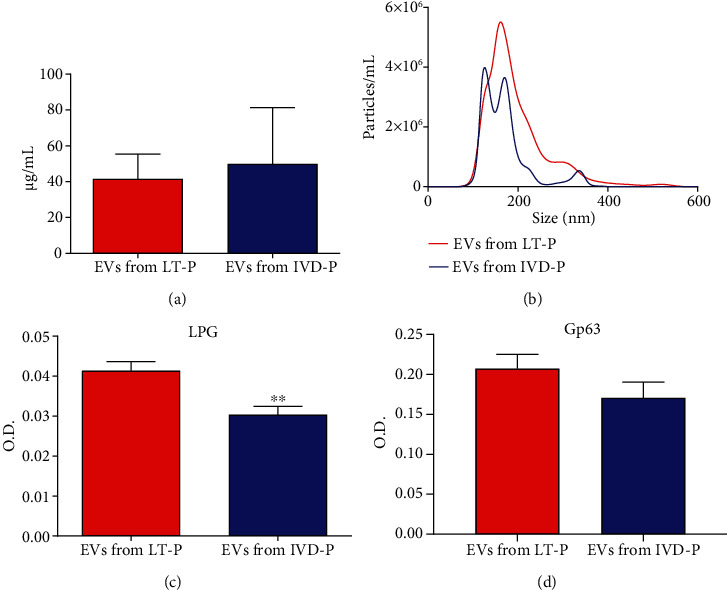
Characterization of EVs released *by L. amazonensis* promastigotes with distinct virulence profiles (IVD-P and LT-P). (a) Protein quantification (*μ*g/mL). Bars represent the average of 3 measurements, and error bars shows the SD. (b) Size profile and concentration (particles/mL) measurements. (c) Detection of LPG and (d) LPG in EVs (4 *μ*g/sample). Bars denote the average of 3 measurements, and error bars show the SD. Unpaired *t*-test ^∗∗^*P* < 0.01.

EVs released by IVD-P and LT-P were obtained according to [20] with parasites incubated at 26°C since this temperature showed better results in immunization protocols (Figure 3). No differences in total protein concentration in EVs from IVD-P and LT-P were detected (Figure 5(a)). Concentration and size distribution of EVs released by IVD-P and LT-P were similar and are shown in Figure 5(b). LPG and gp63 were detected in EVs released by IVD-P and LT-P (Figures 5(c) and 5(d)). However, a significant lower recognition of mAb anti-LPG was observed in EVs released by IVD-P as compared with EVs from LT-P (^∗∗^*P* < 0.01) (Figure 5(c)).

### 3.4. Protective Responses of EVs Released by LT-P or IVD-P

**Figure 6:**
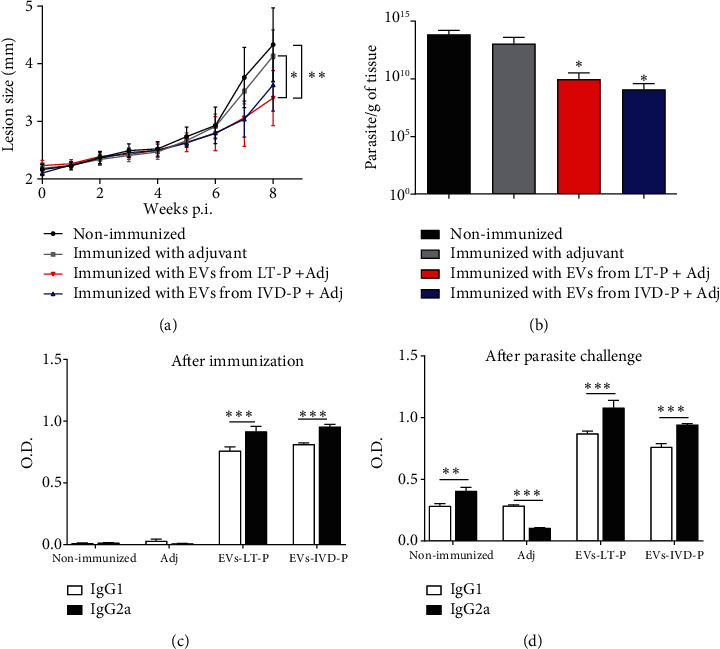
BALB/c mice were managed with the two-dose protocol. (a) Footpad size (millimeter (mm)) measurements (*n* = 7). Each point represents the average of the measurements. ANOVA followed by a post hoc Tukey's test; ^∗^*P* < 0.05: adj compared with mice immunized with LT-P; ^∗∗^*P* < 0.01: nonimmunized infected mice compared with the group immunized with LT-P. (b) Parasite load (*n* = 7). Bars denote the average of measurements, and error bars denote the SD. Kruskal-Wallis test followed by Dunn's test; ^∗^*P* < 0.05 compared with the nonimmunized group. (c) Production of IgG1 and IgG2a isotype antibody anti-EVs after 2 doses of EV immunizations. (d) Measurement of the IgG1 and IgG2a isotype antitotal extract of *L. amazonensis* in mice immunized and challenged with the parasite. Bars represent the average of 7 measurements, and error bars show the SD. Two-way ANOVA followed by post hoc Sidak's test (^∗∗^*P*<0.01 and^∗∗∗^*P* < 0.001 IgG1 versus IgG2a in each group). Nonimmunized: nonimmunized infected mice; Adj: mice immunized with adjuvant; EVs-LT-P: mice immunized with EVs released by LTP-P; EVs-IVD-P: group of mice immunized with EVs from IVD-P. Data are representative of 3 independent experiments.

The possible protective role of EVs released by LT-P or IVD-P parasites was assessed in BALB/c mice immunized intraperitoneally with each EVs. To improve the immune response, EVs were injected in the presence of adjuvant (Adj). Animals received 2 doses of EVs, and after 8 weeks of infection, a significant reduction in lesion size was observed in animals immunized with LT-P compared with the nonimmunized group or mice immunized with Adj alone (^∗^*P* < 0.05;  ^∗∗^*P* < 0.01) (Figure 6(a)). A significant reduction in parasite burden was observed in both groups immunized with EVs (^∗^*P* < 0.05) (Figure 6(b)).

After two doses of EVs, we detected a significant increase in IgG2a levels in comparison with IgG1 (^∗∗∗^*P* < 0.001) (Figure 6(c)). After 8 weeks of infection, animals immunized with EVs maintained higher levels of IgG2a compared with IgG1, except the adjuvant group that showed an increase in IgG1 levels (^∗∗^*P* < 0.01;  ^∗∗∗^*P* < 0.001) (Figure 6(d)). These results suggest a partial protection and an induction of Th1 response in animals immunized with EVs released by LT-P and IVD-P.

**Figure 7:**
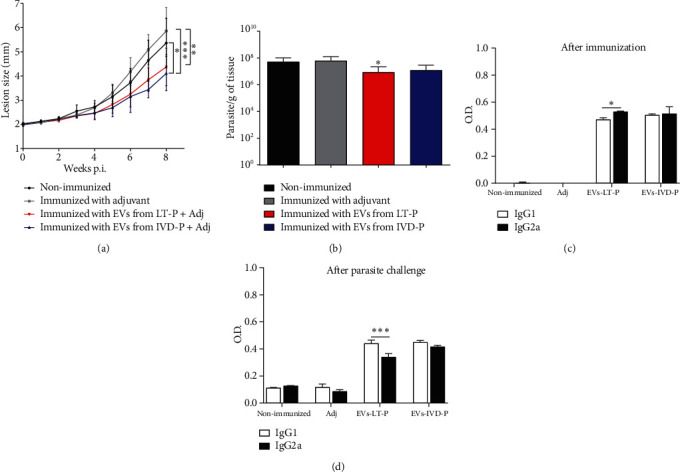
BALB/c mice were treated with the 3-dose protocol. (a) Lesion size (millimeter (mm)) measurements (*n* = 9). Each point represents the average of the measurements. ANOVA followed by post hoc Tukey's test; ^∗^*P* < 0.05: nonimmunized infected mice compared with IVD-P; ^∗∗^*P* < 0.01: adj compared with mice immunized with LT-P; ^∗∗∗^*P* < 0.001: adj compared with the group immunized with IVD-P. (b) Parasite load (*n* = 9). Bars denote the average of 9 measurements, and error bars denote the SD. Kruskal-Wallis test followed by Dunn's test, ^∗^*P* < 0.05 compared with nonimmunized mice. (c) Production of IgG1 and IgG2a isotype antibody anti-EVs. (d) Measurements of the IgG1 and IgG2a isotype anti-total extract of *L. amazonensis* in mice immunized and infected with the parasite. Bars represent the average of 9 measurements, and error bars show the SD. Two-way ANOVA followed by post hoc Sidak's test (^∗^*P*<0.05 and^∗∗∗^*P* < 0.001 IgG1 versus IgG2a in each group). Nonimmunized: nonimmunized infected mice; Adj: mice immunized with adjuvant; EVs-LT-P: mice immunized with EVs released by LTP-P; EVs-IVD-P: group of mice immunized with EVs from IVD-P. Data are representative of 2 independent experiments.

The promising results obtained with 2 immunizations with EVs led us to assess whether an additional dose to the immunization protocol could synergize and led to a more protective effect. Thus, 3 immunizations were performed in the presence of adjuvant with an interval of 2 weeks between doses. After parasite challenge, a significant reduction in lesion size was observed in animals immunized with EVs compared with the nonimmunized or immunized with adjuvant alone groups (^∗^*P* < 0.05;  ^##^*P* < 0.01;  ^∗∗∗^*P* < 0.001) (Figure 7(a)). A significant decrease in parasite load was observed in the group immunized with EVs from LT-P, compared with the nonimmunized group (^∗^*P* < 0.05) (Figure 7(b)).

After 3 doses, a significant increase in IgG2a levels was detected in the group immunized with EVs from LT-P (^∗^*P* < 0.05) (Figure 7(c)). No differences between the levels of IgG1 and IgG2a were observed in animals immunized with EVs from IVD-P (Figure 7(c)). After 8 weeks of challenge with the parasite, animals immunized with EVs from LT-P showed a significant decrease in IgG2a levels, compared with IgG1 levels (^∗∗∗^*P* < 0.001) (Figure 7(d)). Again, no differences were observed between IgG1 and IgG2a in the group immunized with EVs from IVD-P (Figure 7(d)). Our data suggest a mixture of Th1/Th2 responses in animals immunized with 3 doses of EVs from IVD-P and infected with *L. amazonensis* and the Th2 profile in animals immunized with the same immunization scheme with EVs from LT-P.

### 3.5. Cytokine Production by Spleen Cells Isolated from Immunized Mice

**Figure 8:**
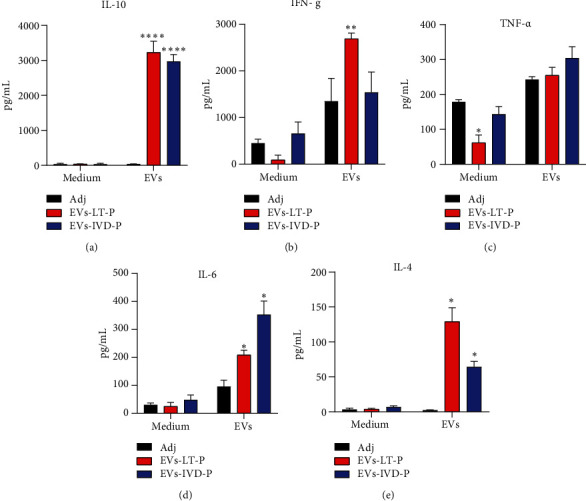
Cytokine responses in mice immunized with EVs from LT-P and IVD-P. BALB/c mice (*n* = 5 per group) were immunized with EVs LT-P and EVs IVD-P mixed with adjuvant. Controls received adjuvant alone (Adj). Two weeks after the last dose, spleen cells were collected and cultured with medium alone (medium) or stimulated with each homologous EVs used for immunization (EVs). (a) Measurement of IL-10, (b) IFN-*γ*, (c) TNF-*α*, (d) IL-6, and (e) IL-4 cytokine levels in cell supernatants. The bars indicate that the mean and the error bars denote the standard deviation of the groups. Statistics were performed using two-way ANOVA, followed by the Tukey post test. ^∗^ indicates significant difference in relation to the groups of mice immunized with EVs and adjuvant alone (^∗^*P* < 0.05,  ^∗∗^*P* < 0.01,  and ^∗∗∗∗^*P* < 0.0001).

The cellular immune response induced after immunization with EVs was evaluated by the cytokine production in splenocytes restimulated *in vitro* with the homologous EVs (i.e., splenocytes from animals immunized with EVs released by LT-P were restimulated *in vitro* with EVs from LT-P). Positive controls were performed with splenocytes from all groups stimulated with concanavalin-A (data not shown). Cytokines were analyzed in animals submitted to the protocol with 2 immunizations because this scheme showed the best results in reducing the parasite load. Analysis of the culture supernatants demonstrated a significant increase in IL-10 (Figure 8(a)) and IFN-*γ* (Figure 8(b)) levels by splenocytes isolated from mice immunized with EVs from LT-P and IVD-P and restimulated *in vitro*, as compared with the adjuvant group (^∗∗^*P* < 0.01;  ^∗∗∗∗^*P* < 0.001). No differences were seen in TNF-*α* levels (Figure 8(c)), but a significant increase in IL-6 (Figure 8(d)) and IL-4 (Figure 8(e)) productions was detected in mice immunized with EVs (^∗^*P* < 0.05). This data means that immunization with 2 doses of EVs induced a Th1-related cytokine (IFN-*γ*) but also promoted an increase in the production of cytokines of the Th2 profile (IL-10 and IL-4).

## 4. Discussion

Immunization with EVs in therapies and vaccination protocols have increased in recent years [26]. Studies have suggested that the effects of EVs on parasite experimental models depend on the disease and the characteristics of the pathogen. BALB/c mice pretreated with EVs released by *Trypanosoma cruzi* and subsequently challenged with the parasite showed cardiac complications, increased amastigote nests, showing that these EVs can contribute to parasite infection [27]. On the other hand, the immunization with EVs from nematode *Trichuris muris* was protective in C57BL/6 mice after parasite challenge. Immunized mice showed a reduction in parasite load and increased levels of IgG1, the protective isotype for extracellular pathogens [28]. Our work showed that BALB/c mice immunized with EVs released by *L. amazonensis* cultivated under distinct conditions led to a modulation of parasite load, antibodies, and cytokines.

First, we investigated the influence of temperature in immunization protocols since the temperature can contribute to the changes in EV releasing and their properties [29]. EVs released by *L. amazonensis* incubated at different temperatures induced different cytokine production by B-1 cells and macrophages [20]. A higher production of IL-6 and IL-10 was detected in macrophages stimulated with EVs from parasites cultured at 26°C [20]. Our results demonstrated that EVs obtained with parasites cultured at 26°C showed a potential protective role in immunization protocols with a significant decrease in lesion size (^∗^*P* < 0.05) and parasite load reduction. The significant increase in the production of specific antibody IgG2a anti-EVs suggests a modulation to a Th1 profile in mice immunized with EVs from parasites incubated at 26°C (^∗^*P* < 0.05;  ^∗∗^*P* < 0.01;  ^∗∗∗∗^*P* < 0.001). The ability to stimulate cytokine production in macrophages may have contributed to the better performance of EVs obtained at 26°C in our immunization protocol [20, 21]. Thus, we included 26°C as the best temperature to obtain EVs for our immunization protocols.

One of the well-known strategies for developing vaccines, including for leishmaniasis, is the use of attenuated parasites by genetic modification or by cultivation for long periods in culture (reviewed in [30]). Immunization studies with these live attenuated parasites have shown promising results against cutaneous leishmaniasis (reviewed in [30]). However, some disadvantages and some issues still need to be better evaluated, such as the behavior of the attenuated parasite in immunocompromised individuals and the possibility of recombination between genetically attenuated and wild-type parasites in the host and/or in the vector [30]. Thus, using EVs from *Leishmania* that can act as vehicles to deliver parasite antigens can be an exciting alternative for immunization protocols. A distinguished feature of *L. amazonensis* EVs for immunization protocols is their higher proinflammatory activity via TLR4/TLR2 [21]. These EVs induced higher levels of NO and cytokines in macrophages (TNF-*α* and IL-6) via TLR4/TLR2 compared with EVs from dermotropic *L. braziliensis* and viscerotropic *L. infantum* [21]. In addition, bone marrow-derived macrophages (BMDM) treated with EVs from *L. amazonensis* had an increase in IL-6 and IL-10 cytokines [20]. This initial proinflammatory effect on macrophages can contribute to the induction of an increase in the Th1 response observed in animals immunized with EVs.

In our work, parasites were cultivated for a long period to obtain isolates with different infectivity. Our data showed that the BALB/c infection with the LT-P decreased the parasite load and reduced the lesion size compared with mice infected with the parasite recovered from the lesion size (IVD-P). Similar studies with *L. infantum* and *L. amazonensis* kept for long periods in culture also reduced parasite infectivity [31, 32]. Although the loss of infectivity has been observed in our study and by [32], attenuation of *L. amazonensis* using the *in vitro* passage was not as evident as demonstrated for other pathogens [33–35]. However, this model offers interesting tools for understanding some mechanisms involved in *Leishmania* infection and studying unknown virulence factors. Furthermore, Magalhães et al. [32] demonstrated that protein expression of *L. amazonensis*-attenuated parasites showed a decrease in molecules related to biological and metabolic functions, infectivity, and motility of the flagellum [32], indicating that this method is capable of causing significant changes in protein expression related to virulence.

EVs are now recognized as new players during parasite-host interaction [16, 19, 36–41]. Given that EVs carry parasite content, we addressed the possibility that EVs from isolates with distinct infectivity could differentially stimulate the immune system. Using immunization protocols with EVs from LT-P or IVD-P, we observed a significant reduction in the parasite load and lesion size compared with nonimmunized animals. However, no significant differences in parasite load were seen between animals immunized with EVs from LT-P and IVD-P. In conclusion, differences in infectivity did not significantly contribute to the protection induced by their respective EVs.

In contrast to our results, previous treatment of mice with EVs from *Leishmania donovani* or EVs from *Leishmania major* led to an exacerbation of infection after a challenge with respective parasites [42]. The ability of *L. amazonensis* EVs to induce an inflammatory response in human and murine macrophages may reinforce their use in immunization protocols [20, 21]. In addition, the presence of LPG and gp63 in EVs from both *L. amazonensis* profiles (LT-P and IVD-P) can partially explain the protection patterns observed. Expression of LPG was lower in IVD-P than in the LT-P resulting in functional differences in splenocyte stimulation. For example, LT-P (high gp63 and high LPG) induced higher levels of IFN-*γ* whereas those from IDV-P (high gp63 and low LPG) induced higher levels of IL-6 and IL-4. A previous work showed that LPG from *L. amazonensis* (strain BH125) was able to induce the NO, TNF-*α*, and IL-6 productions by peritoneal murine macrophages [43]. Thus, this molecule can contribute to an initial inflammatory response in immunization, impacting the course of the experimental infection. Besides gp63 and LPG, *Leishmania* EVs carry different parasite antigens that can stimulate the immune response [44–47]. Proteomic studies demonstrated the presence of some important virulence factors in EVs released by *Leishmania*, such as cysteine peptidase, EF-1 alpha, enolase, HSP70, and peroxidoxin [47]. The presence of these molecules can also influence the stimulation of Th1/Th2 response profiles observed in our immunization model. Thus, proteomic analyzes of LT-P and IVD-P EVs may help understand possible mechanisms involved in the immune response against EVs detected in our immunization protocols.

Immunization with *L. amazonensis* EVs showed a potential protective role with a decrease in parasite load after two or three doses. However, a more pronounced decrease (reduction in approximately 4 logs) in parasite load in animals immunized with two doses of EVs was observed. Immunization with two doses induced higher IFN-*γ* levels by splenocytes, a cytokine involved with the protective response. A higher level of IgG2a antibodies with two immunizations was also detected before and after the challenge with the parasite, suggesting an induction of Th1 profile using this protocol. Although the presence of IL-10 and IL-4 had increased, the presence of proinflammatory cytokines (IL-6 and IFN-*γ*) may be related to the lower parasite load observed in animals immunized with two doses. Two hypotheses can be speculated to better performance with two doses: the alum adjuvant and the EV constitution. The alum adjuvant is known to stimulate the Th2 response. So, the addition of one more dose could have stimulated the Th2 profile, related to a no protective response. Some constituents present in EVs also could be modulating the Th2 response. These important questions are under investigation in our laboratory.

Based on literature, the dose and immune response relationship in vaccines is not fully understood and lower doses have been somehow more immunogenic than higher ones (reviewed in [48]). For example, in a preclinical study with tuberculosis vaccines, this phenomenon was reported [49]. The higher immunogenicity to tuberculosis antigens was seen with doses from 5 to 15 *μ*g of antigens, but there was a decrease in this parameter with 50 and 150 *μ*g [49]. In addition, the type of immunization regimen has an impact on protection. A study using a vaccine prototype against malaria showed that the use of fractionated doses and the dose spacing significantly increased the protection against infection [50]. Therefore, our study contributed to this proposal that it is important to consider studying the vaccine prototype application regime under a new perspective to better understand the immunological response in vaccine models.

Altogether, our data revealed the potential protective effects of EVs released by *L. amazonensis* in an experimental cutaneous leishmaniasis model. Although the immunization with EVs showed a partial effect on eliminating the parasite, the promising results obtained with two immunizations led to a lower parasite load and polarization response to the Th1 profile in animals challenged with the parasites. Changes in the protocols including new adjuvants and/or parasite strains may improve such responses. Some publications demonstrated promising results using vaccine preparations with *Leishmania* total antigens [8, 51]. *Leishmania* EVs are not incorporated in those protocols since the parasites are often washed to obtain the extracts. The incorporation of EVs into these preparations could lead to an additional protective benefit. Thus, our work can bring new and interesting possibilities for studying EVs in leishmaniasis as possible candidates for immunization protocols.

## 5. Conclusions

In this work, EVs from *L. amazonensis* cultured under different temperatures and EVs from parasites with different infectivity were produced and characterized. These EVs showed potential a protective role in the immunization model, inducing the production of Th-1 related cytokine and specific antibodies against the parasites. Furthermore, the long-term *in vitro* culture did not change the ability to induce partial protection in experimental cutaneous leishmaniasis. Our results are aimed at contributing to a better understanding of the role of EVs in the parasite-host interaction and validating a protective approach in an immunization model with parasite antigens.

## Data Availability

Data supporting the findings are available from the corresponding author upon request.
